# Morin Ameliorates Lipopolysaccharides-Induced Sepsis-Associated Encephalopathy and Cognitive Impairment in Albino Mice

**DOI:** 10.1007/s11064-024-04269-8

**Published:** 2024-11-16

**Authors:** Asmaa R. Mohamed, Nagui H. Fares, Yomna I. Mahmoud

**Affiliations:** Zoology Department, Faculty of Science, Ain Shams University, Cairo, Egypt

**Keywords:** Sepsis-associated encephalopathy, Lipopolysaccharides, Morin, Neuroprotective, Behavior, Mice

## Abstract

Sepsis-associated encephalopathy is a common neurological complication of sepsis that is characterized by neuroinflammation, oxidative stress and apoptosis, which results in cognitive impairments in septic survivors. Despite numerous treatment options for this condition, none of them are definite. Therefore, this study aimed to investigate the impact of morin, a flavone known for its neuroprotective and anti-inflammatory effects, against lipopolysaccharides-induced sepsis-associated encephalopathy in albino mice for 7 days. Mice were divided into 4 groups: Negative control, morin, septic, and septic morin-treated mice. Sepsis was induced by a single injection of lipopolysaccharides (5 mg/kg, intraperitoneally), morin (50 mg/kg b. wt.) was given orally, starting from 5 h after sepsis induction, then daily for 4 other days. Morin ameliorated septic structural and functional alternations as manifested by improving the survival rate, the behavioral functions, in addition to preserving and protecting the brain tissue. This was accompanied with the augmentation of the total antioxidant capacity, as well as the suppression of tissue levels of the lipid peroxidation marker malondialdehyde, apoptosis (cleaved-caspase-3), glial fibrillary acidic protein, and the proinflammatory cytokine tumor necrosis factor. In conclusion, morin has a promising ameliorative effect to counteract the sepsis-associated encephalopathy via its anti-inflammatory and antioxidant effects and to prevent the associated cognitive impairments.

## Introduction

Sepsis-associated encephalopathy (SAE) frequently occurs as a neurological complication of sepsis. It manifests as widespread brain dysfunction and cognitive impairment resulting from an infection originating elsewhere in the body [1]. The pathophysiology of SAE is complex, involving interconnected processes that result in various functional alterations and disorders, including neuroinflammation, oxidative stress, and impaired integrity of the blood-brain barrier [2]. Additionally, peripheral cytokine storm triggers the release of inflammatory mediators, primarily marked by microglial activation, leukocyte infiltration, and neuronal degeneration. Simultaneously, there is an elevation in the permeability of the blood-brain barrier [3] facilitating the entry of peripheral inflammatory mediators into the central nervous system, intensifying blood-brain barrier permeability, and amplifying the generation of inflammatory mediators [4]. Microglia respond rapidly by producing significant amounts of nitric oxide, TNF-α, oxygen-free radicals, and various excitatory neurotransmitters. This expanded inflammatory cascade exacerbates neuronal damage, consequently impairing states of consciousness and emotional states [5]. Clinical manifestations of SAE can vary, encompassing mild symptoms like malaise and concentration deficits to severe states, such as deep coma. Despite the availability of numerous treatment options, there is currently no conclusive therapy for SAE. As a result, there remains a critical need for potential treatments that could alleviate the effects of SAE [2]. Morin is a bioactive compound present in various fruits and herbs [6]. It is a nontoxic compound with diverse pharmacological activities, including antimicrobial, antioxidant, anti-inflammatory, and neuro-protective properties [7]. The decision to utilize morin in the treatment of SAE in this study was based on former studies that demonstrated its neuroprotective effects in various neurodegenerative diseases such as Alzheimer’s disease [8], diabetic neuropathy [9], Parkinson’s disease [10], and ischemic stroke [11], in addition to ifosfamide-induced neurotoxicity and acute stress-induced behavioral changes [12, 13]. Moreover, both in vitro and in vivo studies indicate that morin has very low toxicity and is well-tolerated with chronic use [14]. Therefore, the purpose of this study is to explore the role of morin against SAE, and to investigate the mechanism of its action.

## Materials and methods

### Animals

Eight-week-old male CD-1 strain mice, weighing 25 to 30 g, were purchased from the Medical Research Centre at the Faculty of Medicine, Ain Shams University (Cairo, Egypt). All animal experiments were approved and carried out according to the Research Committee of Zoology Department, Ain Shams University (12/04/2021-5). Mice were housed under hygienic conditions in polypropylene cages and acclimatized to laboratory conditions for a period of 1 week before the commencement of the experiments to eliminate the effect of stress. Animals were kept under good ventilation under a photoperiod of 12 h light: 12 h dark. A temperature of 23–25 °C and 45–55% relative humidity were maintained. Due to the impact of estrogen levels and the physiological cycle on anxiety-like behavior, male mice were used in this experiment [15].

## Procedures

### Drugs

Lipopolysaccharides (LPS) (From *Escherichia coli* 0111:BA; cat# L2630) with purity > 96.5%, and morin hydrate (cat# M4008) with purity > 85.8% were acquired from Sigma-Aldrich Company (USA). Both LPS and morin were dissolved in normal saline and freshly-prepared before use.

### Experimental Protocol and Design

Sixty animals were randomly assigned into four groups as follows:

- **Group I** (Negative control group, *n* = 10): normal mice, received a single intraperitoneal injection of normal saline (5 mg/kg), and normal saline (50 mg/mice, orally) for 5 days.
- **Group II** (Morin group, *n* = 10): mice were orally treated with morin hydrate at a daily dose of 50 mg/kg b.wt for 5 consecutive days.
- **Group III** (Septic group, *n* = 20): LPS-injected mice. To establish SAE model, mice were intraperitoneally injected with a single LPS injection, at a dose of 5 mg/kg in PBS.
- **Group IV** (Septic morin-treated group, *n* = 20): LPS-injected mice, then were orally treated with morin hydrate (50 mg/kg b.wt) for 5 days.

Mice in all groups received the vehicles of LPS and morin group at the same volume and route. LPS dose for inducing sepsis-associated encephalopathy and the dose of morin for treatment in animal models were also chosen according to previous studies [16, 17], respectively. The clinical symptoms of all mice were observed and the survival rate was monitored over the 7 days of the experiment.

During days 2 to 6, some standard behavioral tests were carried out, such as elevated plus maze (on day 2), open field test (on day 3), novel object recognition (on day 4, 5), tail suspension test (on day 5), and T-maze test (on day 6). These tests were applied to evaluate mood disturbances (anxiety and depression) and motor disorder in all experimental groups. On day 7, animals were fasted overnight, weighed, then sacrificed by cervical dislocation and the brains were quickly excised, washed in saline, and blotted dry. The brains were then divided in 2 parts for biochemical analysis and histological assays.

## Behavioral Methods

Behavioral tests were conducted on 7 animals from each group. These tests were administered in order, starting from less stressful to more stressful conditions from day 2 to day 6 after induction with LPS.

### The Elevated plus Maze Test

The elevated plus-maze test was used to assess anxiety-related behaviors in mice. The procedure was performed according to a previously reported protocol of [18] with some modifications. 24 h after LPS injection, the animals were positioned individually in the center zone of the elevated plus maze (10 cm × 10 cm), oriented towards one of the open arms (50 cm above the floor). Over a 5-minute period, the number of entries and duration spent in both the open arms (50 cm × 10 cm) and enclosed arms (50 cm × 10 cm, with 40 cm-high walls) were meticulously recorded. The percentages of open arm entries [open/total entries×100] and time spent in open arms [open / (open + closed-arm time) ×100] were calculated for each animal. The heightened activity in the open arms (entry and time) indicates a reduction in anxiety-related behaviors. Additionally, the total entries (sum of the entries into the open and closed arms) were analyzed as an indicator of locomotor activity.

### The Open Field Test

The open field test was carried out to assess the motor performance and non-associated memory. The procedure was performed according to the previously reported protocol of [19] with some modifications. The apparatus used was a square woody box of (50 × 50 × 40 cm), and the ground of the open field was divided by black lines into 25 equal squares. Two days post-sepsis induction, mice were gently introduced into the center of the box for a 5-minute exploration period. Subsequently, the animals were returned to their home cage. Various parameters, including the total distance, total time traveled, center square entries, and line crossing in the open field were meticulously recorded. Elevated frequencies of these behaviors indicated increased locomotion and exploration and/or a reduced level of anxiety Following each trial, the arena was cleaned with 75% alcohol to avoid the presence of olfactory cues.

### The Novel Object Recognition Test

The novel object recognition test was carried out to assess short-term memory. The procedure was performed according to the previously reported protocol of [20] with some modifications. The experimental protocol consisted of three sessions: habituation, training, and testing. Each mouse was placed in a woody cage (50 cm ×50 cm ×50 cm). Habituation session was carried out for 5 min on the first day when the mouse was introduced into empty chambers without any object. Twenty-four hours later, the mouse entered the training session. In this phase, the mouse was positioned in the same chamber facing the wall opposite to two identical objects (A1 and A2) and was allowed to explore the objects for 10 min. The time spent exploring each object was recorded. After a delay of 24 h, the mouse was returned to the chamber with one of the familiar objects (A2) replaced by a novel object (A3). During this session, mouse freely explored the familiar object (A1) and the new object (A3) for 10 min, and exploration times for each object were recorded. The exploration time for each object during the test session was recorded. The discrimination ratio was calculated as follows: time of exploration of the novel object/ (time of exploration of the familiar object + time of exploration of the novel object) ×100. An index greater than 0.5 indicates a preference for the novel object. To control for olfactory cues, the apparatus and objects were meticulously cleaned with 75% ethanol between each trial.

### The Tail Suspension Test

Tail suspension test was conducted according to the previous study of [21] with some modifications. Each mouse’s tail was affixed with adhesive tape and suspended at a height of 50 cm. The duration of immobility during the 6 min test period was recorded and the immobility time was precisely measured over a last 4-minute interval.

### The T-maze Test

Working memory was assessed using the T-maze test. The procedure was performed according to a previously reported protocol of [22] with some modifications. The apparatus was crafted from wood and consisted of a wooden platform (length of start and goal stems = 75 cm, width = 12 cm, height = 20 cm). The guillotine doors and the floor of the apparatus were colored black. The training session for each mouse encompassed a single session, commenced with one forced-choice trial succeeded by 14 free-choice trials. In the initial trial, the ‘forced-choice trial’, either the left or right goal arm was blocked by lowering the guillotine door. After the mouse was released from the start arm, it negotiated the maze, entered the open goal arm, and returned to the start position. There, the animal was confined for 5 s by lowering the guillotine door of the start arm. During 14 ‘free-choice’ trials, the mouse had the liberty to choose between the left and right goal arms. Upon opening the guillotine door of the start arm the animal was free to choose between both goal arms (all guillotine doors open). Once the mouse entered one goal arm, the other goal arm was closed. The mouse then returned to the start arm, and the next free-choice trial started after a 5-seconds confinement in the start arm. A session was terminated and the animal once either 14 free-choice trials were completed or 30 min were elapsed, whichever occurred first. To isolate the cognitive component, care was taken to diminish the olfactory cues present by cleaning the maze with a damp sponge before each new animal started its session, as these cues can influence the alternation rate.

### Biochemical Assays

The levels of the malondialdehyde (MDA) and total antioxidant capacity (TAC) in hippocampus tissues were determined using enzyme-linked immunosorbent assay (ELISA). The values were reported as n.mol/mg of protein for MDA, and ng/mg of protein for TAC.

### Histology

Cerebral cortex and hippocampus slices were harvested for histological investigation. After 24 h, brain samples were left to dry at room temperature in a solution of 10% neutral buffered formalin. Subsequently, the samples underwent an overnight rinsing process in running tap water. They were subjected to a progressive series of alcohol dehydration, terpineol clearing, and embedding in paraffin wax to produce 5-mm thick sections, were then dewaxed, hydrated, and stained with hematoxylin and eosin for light microscopy according to [23]. The number of apoptotic cells was calculated as the area ratio of the apoptotic cells to the image. The outcomes from each section were averaged and subjected to statistical analysis using ImageJ software (Melville, NY, USA).

### Immunohistochemistry

Paraffin sections for GFAP (which increases in neurodegenerative diseases), pro-inflammatory cytokines TNF-α, and cleaved caspase-3 (for apoptosis) immune-expression were performed using avidin–biotin–peroxidase technique [24]. The area of protein expression was calculated as the percentage of the image area occupied by protein expression. The outcomes from each section were averaged and subjected to statistical analyses using ImageJ software (Melville, NY, USA).

### Statistical Analysis

All statistical analyses were conducted using IBM SPSS Statistics version 26 (IBM Corp., Armonk, NY, USA) and Minitab version 19 (Minitab LLC, State College, PA, USA). Data were expressed as mean ± standard deviation (SD). Descriptive statistics were used to summarize the data across the experimental groups. Data normality was assessed using the Shapiro-Wilk test for each experimental group. If data were normally distributed (*p*-value > 0.05), homogeneity of variances was verified using Levene’s test, and a two-way ANOVA was used to evaluate the effects of Morin, LPS, and their interaction. Post-hoc comparisons were conducted using Bonferroni adjustments. A *p*-value of less than 0.05 was considered statistically significant. Otherwise, Kruskal-Wallis test was applied to determine differences among groups, with pairwise comparisons adjusted using Bonferroni corrections. Graphical representations of the data, including scatter plots, were generated using Microsoft Excel 2019 (Microsoft Corp., Redmond, WA, USA) to visualize the findings.

## Results

### Behavior Tests

#### Elevated plus Maze Test

**Fig. 1:**
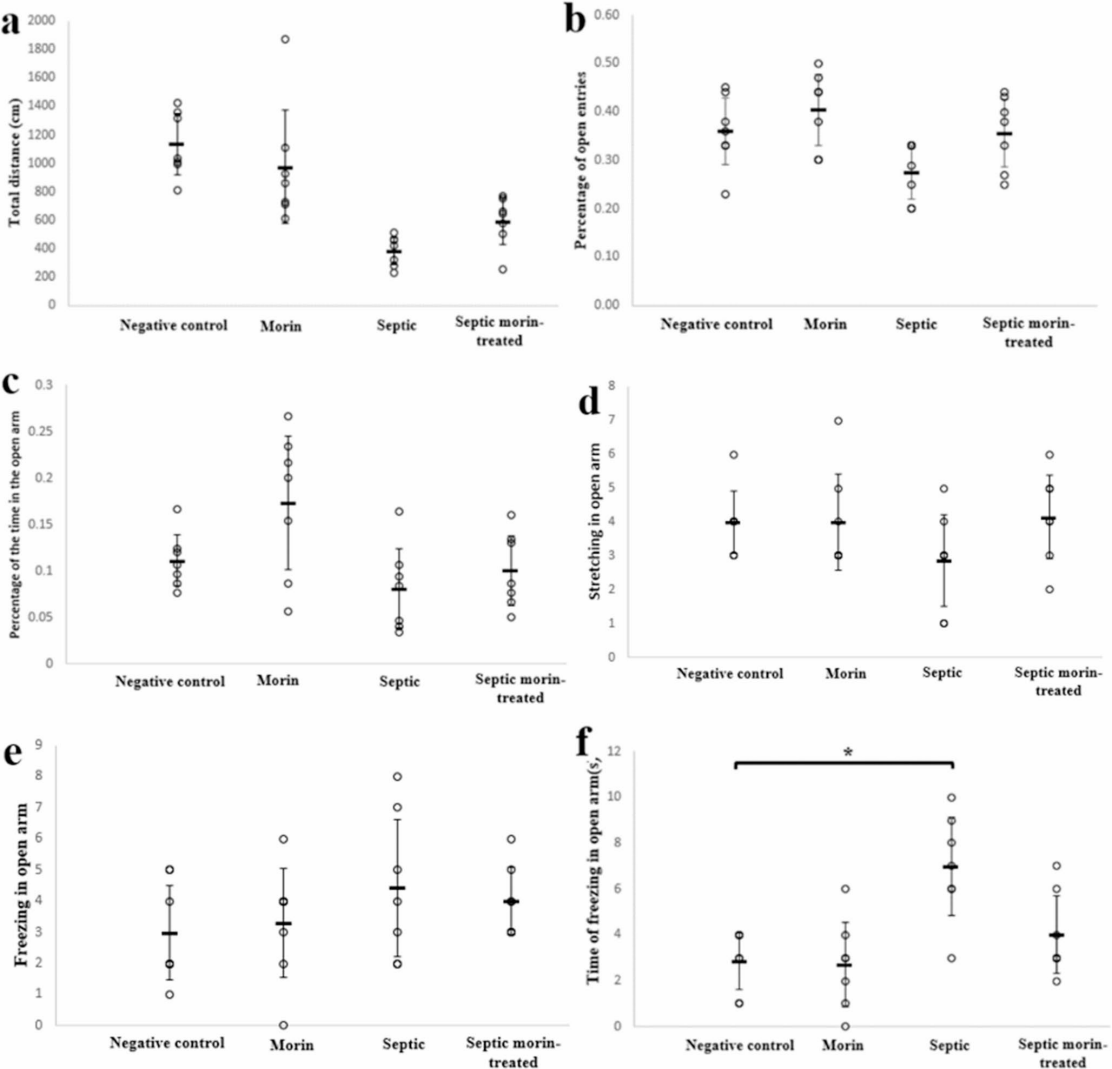
(**a**) Total distance (cm), (**b**) percentage of open entries, (**c**) percentage of the time in the open arm, (**d**) stretching in open arm, (**e**) freezing in open arm, and (**f**) time of freezing in open arm (s) in negative control group, morin group, septic group, septic morin-treated group in elevated plus maze test in mice. All data are expressed as mean ± SD (*n* = 7). Circles represent raw data; horizontal lines represent mean for each group. (*) Symbol represents significance compared with negative control group, where (*): *p* < 0.05 and (**): *p* < 0.001. (#) Symbol represents significance compared with septic group, where (#): *p* < 0.05 and (# #): *p* < 0.001

The elevated plus maze test was conducted to assess the effect of morin on locomotor activity, exploration, and anxiety. 2-way ANOVA revealed no significant interaction between morin and LPS on total distance traveled (*F*_1,24_ = 3.99, *p* > 0.05) (Fig. 1a). Similarly, no significant interaction between morin and LPS on the percentage of open-arm entries was observed (*F*_1,24_ = 0.45, *p* > 0.05) (Fig. 1b). Additionally, the analysis showed no significant interaction between sepsis and morin interaction on the percentage of time in the open arm (*F*_1,24_ = 1.20, *p* > 0.05) (Fig. 1c). The Kruskal-Wallis test also indicated no significant differences in stretching in the open arm between LPS and morin treatment groups (*H*_3_ = 3.439, *p* > 0.05) (Fig. 1d). Furthermore, 2-way ANOVA revealed no significant interaction on freezing in the open arm (*F*_1,24_ = 0.27, *p* > 0.05) (Fig. 1e). For the time of freezing in the open arm, the Kruskal-Wallis test indicated significant differences between the groups (*H*_3_ = 10.733, *p* < 0.05). After Bonferroni correction, pairwise comparisons revealed that the LPS group had a significant effect on the time of freezing in the open arm compared with the negative control group (adjusted *p* < 0.05), while the septic morin-treated group exhibited a non-significant effect on the time of freezing in the open arm compared with LPS (adjusted *p* > 0.05) (Fig. 1f).

#### Open Field Test

**Fig. 2:**
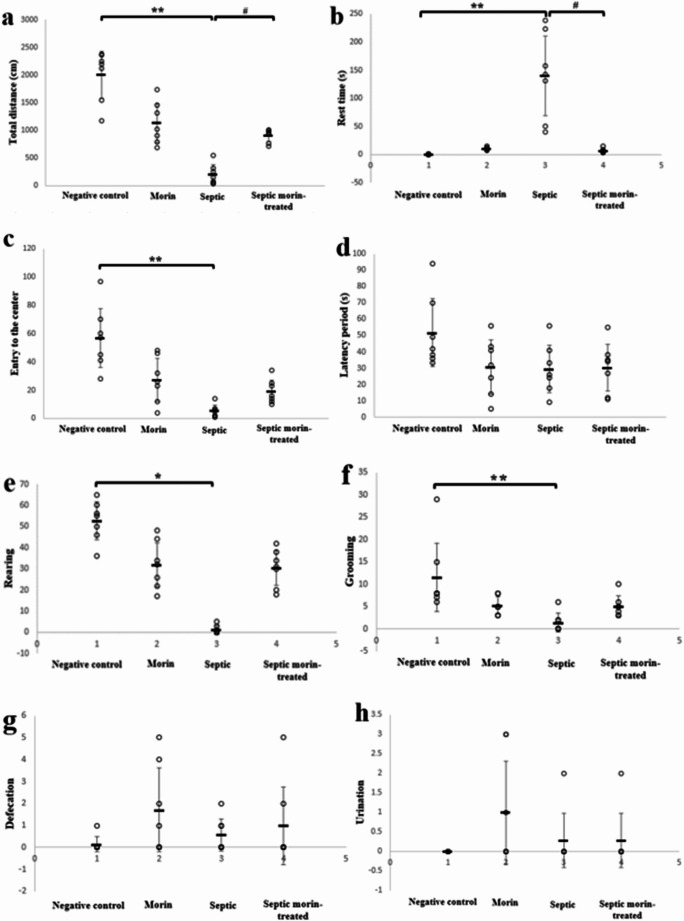
(**a**) Total distance (cm), (**b**) Rest time (s), (**c**) entry to the center, (**d**) latency period (s), (**e**) rearing, (**f**) grooming, (**g**) defecation, and (**h**) urination in negative control group, morin group, septic group, septic morin-treated group in open field test in mice. All data are expressed as mean ± SD (*n* = 7). Circles represent raw data; horizontal lines represent mean for each group. (*) Symbol represents significance compared with negative control group, where (*): *p* < 0.05 and (**): *p* < 0.001. (#) Symbol represents significance compared with septic group, where (#): *p* < 0.05 and (# #): *p* < 0.001

The open field test was performed to evaluate the effects of morin on locomotor activity, exploratory behavior, and anxiety. 2-way ANOVA revealed a significant interaction between morin and LPS for total distance traveled (*F*_1,24_ = 42.44, *p* < 0.001). Post-hoc pairwise comparisons, using Bonferroni correction, showed that LPS significantly reduced total distance compared with the negative control group (Bonferroni-adjusted *p* < 0.001), while the septic morin-treated group had a significant effect on total distance compared with the LPS group (Bonferroni-adjusted *p* < 0.05) (Fig. 2a). For rest time, the Kruskal-Wallis test indicated significant differences between the groups (*H*_3_ = 23.70, *p* < 0.001). After Bonferroni correction, pairwise comparisons revealed that the LPS group had significantly increased rest time compared with the negative control group (adjusted *p* < 0.001), while the septic morin-treated group exhibited a significantly reduced rest time compared with the LPS group (adjusted *p* < 0.05) (Fig. 2b). Furthermore, 2-way ANOVA also showed a significant interaction between morin and LPS for entries to the center (*F*_1,24_ = 15.30, *p* < 0.05). Post-hoc analyses, using Bonferroni correction, revealed that LPS significantly reduced entries to the center compared with the negative control group (Bonferroni-adjusted *p* < 0.001), while the septic morin-treated group did not significantly affect this behavior compared with the LPS group (Bonferroni-adjusted *p* > 0.05) (Fig. 2c). 2-way ANOVA showed no significant interaction between LPS and morin in terms of latency period (*F*_1,24_ = 2.553, *p* > 0.05) (Fig. 2d). For rearing behavior, the Kruskal-Wallis test indicated significant differences among groups (*H*_3_ = 21.36, *p* < 0.001). After Bonferroni correction, pairwise comparisons revealed that the LPS group had a significantly reduced rearing behavior compared with the negative control group (adjusted *p* < 0.05), while the septic morin-treated group did not significantly improve rearing behavior compared with the LPS group (adjusted *p* > 0.05) (Fig. 2e). Additionally, the Kruskal-Wallis test showed significant differences among groups in grooming behavior (*H*_3_ = 16.295, *p* < 0.05). After Bonferroni correction, pairwise comparisons revealed that the LPS group showed significantly increased grooming compared with the negative control group (adjusted *p* < 0.001), whereas the septic morin-treated group did not significantly affect grooming in comparison with the LPS group (adjusted *p* > 0.05) (Fig. 2f). Finally, the Kruskal-Wallis test revealed no significant differences in defecation (*H*_3_ = 3.495, *p* > 0.05) (Fig. 2g), or urination (*H*_3_ = 4.684, *p* > 0.05) (Fig. 2h).

#### Novel Object Recognition Test

**Fig. 3:**
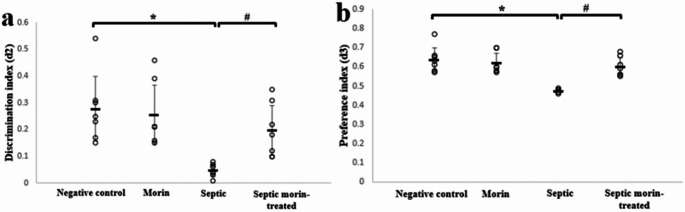
(**a**) Discrimination index, and (**b**) preference index in negative control group, morin group, septic group, septic morin-treated group in novel object recognition test in mice. All data are expressed as mean ± SD (*n* = 7). Circles represent raw data; horizontal lines represent mean for each group. (*) Symbol represents significance compared with negative control group, where (*): *p* < 0.05 and (**): *p* < 0.001. (#) Symbol represents significance compared with septic group, where (#): *p* < 0.05 and (# #): *p* < 0.001

The novel object recognition test was conducted to evaluate the effect of morin on the learning and recognition memory of septic mice. For discrimination index, the Kruskal-Wallis test indicated significant differences between groups (*H*_3_ = 16.24, *p* < 0.001). After applying the Bonferroni correction, pairwise comparisons revealed that the LPS group had a significantly lower discrimination index compared with the negative control group (adjusted *p* < 0.05). Additionally, the septic morin-treated group showed a trend towards significance compared with the LPS group (adjusted *p* < 0.05) (Fig. 3a). Furthermore, the Kruskal-Wallis test indicated significant differences between groups on the reference index (*H*_3_ = 16.190, *p* < 0.001). After Bonferroni correction, pairwise comparisons revealed a significant effect of LPS on the reference index compared with the negative control group (adjusted *p* < 0.05). The septic morin-treated group also showed a significant improvement compared with the LPS group (adjusted *p* < 0.05) (Fig. 3b).

#### Tail Suspension Test

**Fig. 4:**
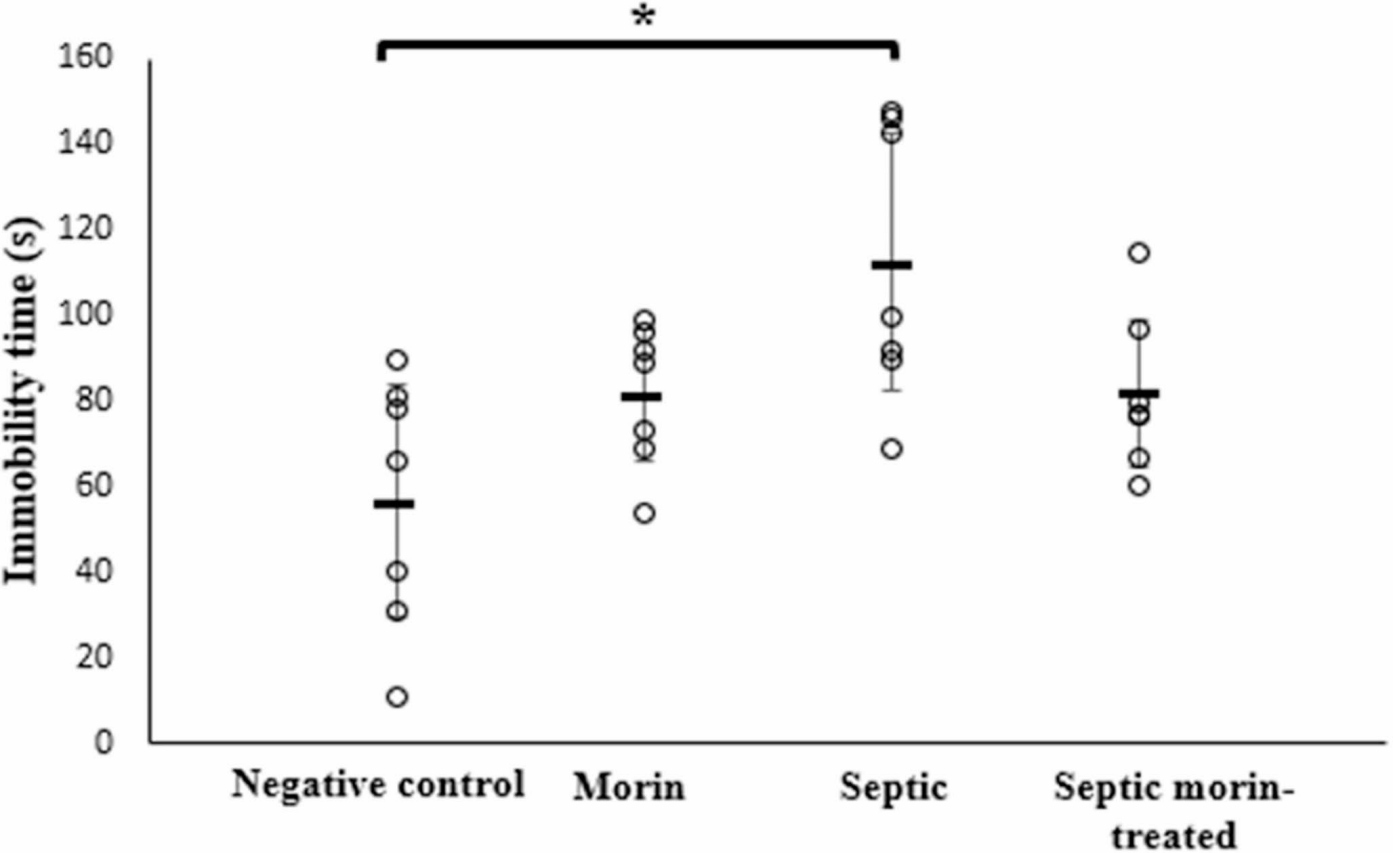
Duration of immobility (s) in negative control group, morin group, septic group, septic morin-treated group in tail suspension test in mice. All data are expressed as mean ± SD (*n* = 7). Circles represent raw data; horizontal lines represent mean for each group. (*) Symbol represents significance compared with negative control group, where (*): *p* < 0.05 and (**): *p* < 0.001. (#) Symbol represents significance compared with septic group, where (#): *p* < 0.05 and (# #): *p* < 0.001

The tail suspension test was used to evaluate the antidepressant-like effects of morin treatment. 2-way ANOVA revealed a significant interaction between morin treatment and LPS on immobility time (*F*_1,24_ = 8.52, *p* < 0.05). Post-hoc pairwise comparisons, with Bonferroni correction, showed that LPS significantly increased immobility time compared with the negative control group (Bonferroni-adjusted *p* < 0.05). However, the septic morin-treated group did not show a significant difference in immobility time compared with the LPS group (Bonferroni-adjusted *p* > 0.05) (Fig. 4).

#### T-Maze Test

**Fig. 5:**
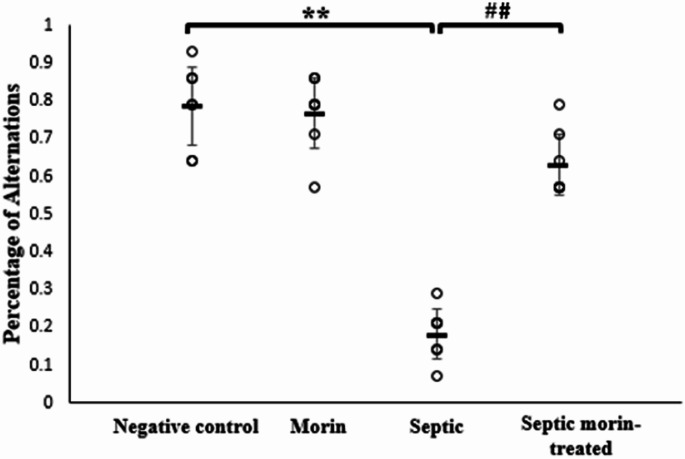
Percent alteration in negative control group, morin group, septic group, septic morin-treated group in T-maze test in mice. All data are expressed as mean ± SD (*n* = 7). Circles represent raw data; horizontal lines represent mean for each group. (*) Symbol represents significance compared with negative control group, where (*): *p* < 0.05 and (**): *p* < 0.001. (#) Symbol represents significance compared with septic group, where (#): *p* < 0.05 and (# #): *p* < 0.001

The T-maze test was employed to evaluate the impact of morin on spatial learning and memory. 2-way ANOVA revealed a significant interaction between morin treatment and LPS on the percentage of alternations (*F*_1,24_ = 43.75, *p* < 0.001). Post-hoc pairwise comparisons, with Bonferroni correction, showed that LPS significantly decreased the percentage of alternations compared with the negative control group (Bonferroni-adjusted *p* < 0.001). Additionally, the septic morin-treated group showed a significant increase in the percentage of alternations compared with the LPS group (Bonferroni-adjusted *p* < 0.001) (Fig. 5).

### Clinical Symptoms

During the whole experiment, the negative control and morin mice appeared healthy and active. In the first 3 days, septic mice showed symptoms of lethargy and hypo-activity appeared in all mice, while diarrhea, limp tail, hind leg paralysis, tetra-paresis and moribund were detected in a few mice. Most of the mice suffered from closed eyes, and slightly ruffled fur, in addition to musty smell. Some mice showed a circling behavior and dizziness during open field test. On the other hand, septic morin-treated mice showed, slightly, the clinical symptoms of the septic mice, such as diarrhea, complete closed eyes and ruffled fur. During the next few days, these clinical symptoms of the surviving mice faded and their activity improved.

### Survival Rate

**Fig. 6:**
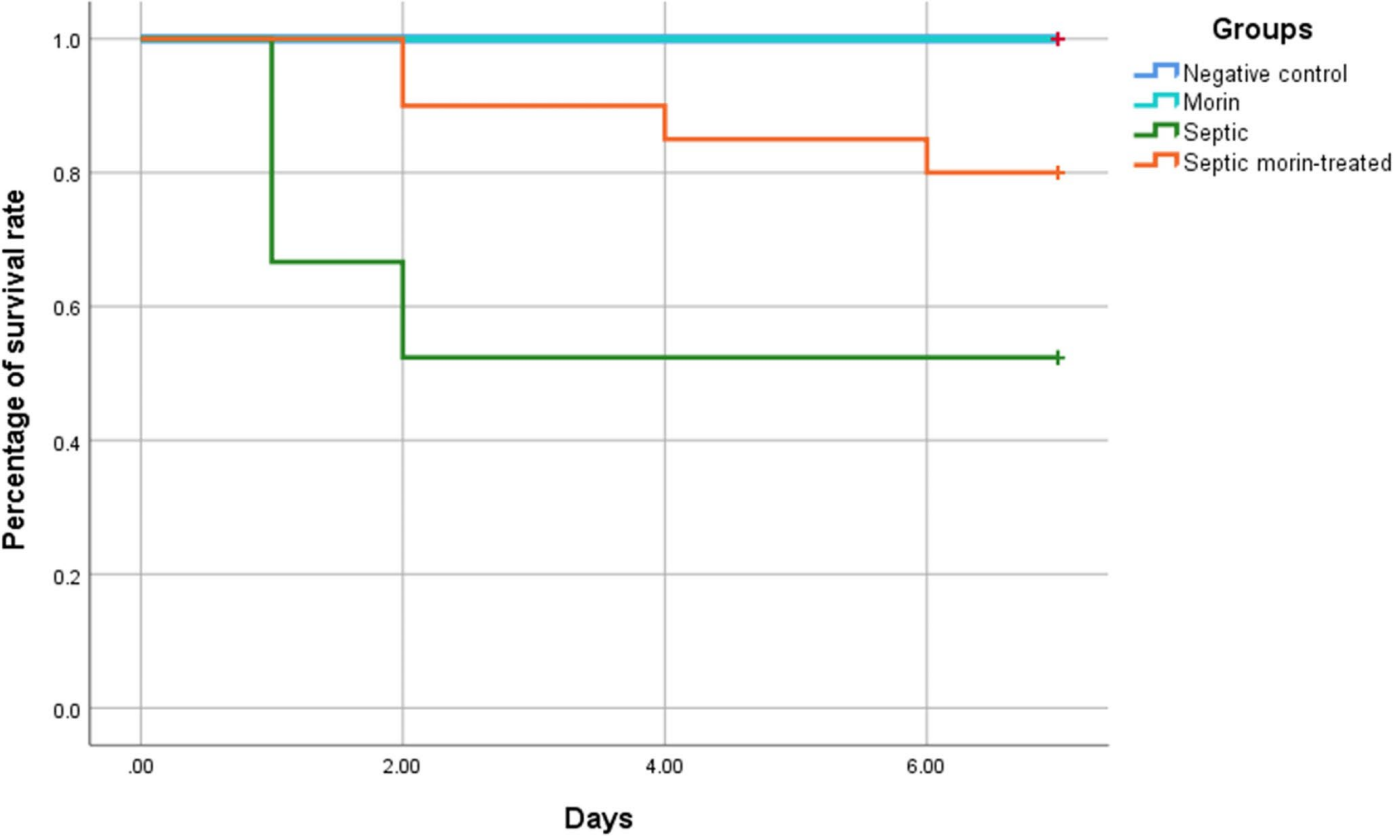
Effect of morin on the survival rate in normal and septic mice

No deaths were recorded in the negative control or morin groups during the 7 days of the experiment (survival rate = 100%). Septic mice, either treated with morin or not, had a lower survival rate versus the negative control. The survival rate of septic group was significantly lower compared with other groups, and rated 52.4%. Treating septic animals with morin increased the survival rate of mice to 80%. Kaplan-Meier survival plots are represented in Fig. 6.

### Biochemical Assays

**Fig. 7:**
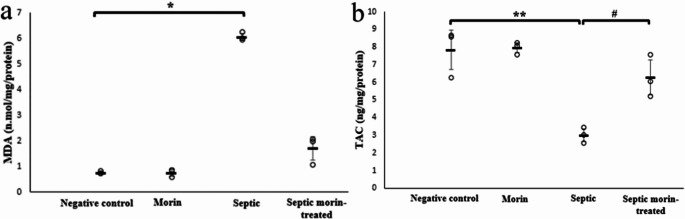
(**a**) MDA (n.mol/ mg hippocampus tissue protein), and (**b**) TAC (ng/ mg hippocampus tissue protein) in negative control group, morin group, septic group, septic morin-treated group of mice. All data are expressed as mean ± SD. Circles represent raw data; horizontal lines represent mean for each group. (*) Symbol represents significance compared with negative control group, where (*): *p* < 0.05 and (**): *p* < 0.001. (#) Symbol represents significance compared with septic group, where (#): *p* < 0.05 and (# #): *p* < 0.001

For MDA, the Kruskal-Wallis test indicated significant differences between groups (*H*_3_ = 11.00, *p* < 0.05). After applying the Bonferroni correction, pairwise comparisons revealed that the LPS group had a significantly increased MDA levels compared with the negative control group (adjusted *p* < 0.05). However, the septic morin-treated group did not show a significance compared with the LPS group (adjusted *p* > 0.05) (Fig. 7a). 2-way ANOVA revealed a significant interaction between morin treatment and LPS on TAC levels (*F*_1,24_ = 8.76, *p* < 0.05). Post-hoc pairwise comparisons, with Bonferroni correction, showed that LPS significantly decreased TAC levels compared with the negative control group (Bonferroni-adjusted *p* < 0.001). Additionally, the septic morin-treated group exhibited a significant increase in TAC levels compared with the LPS group (Bonferroni-adjusted *p* < 0.05) (Fig. 7b).

### Histology

#### Cerebral Cortex

**Fig. 8:**
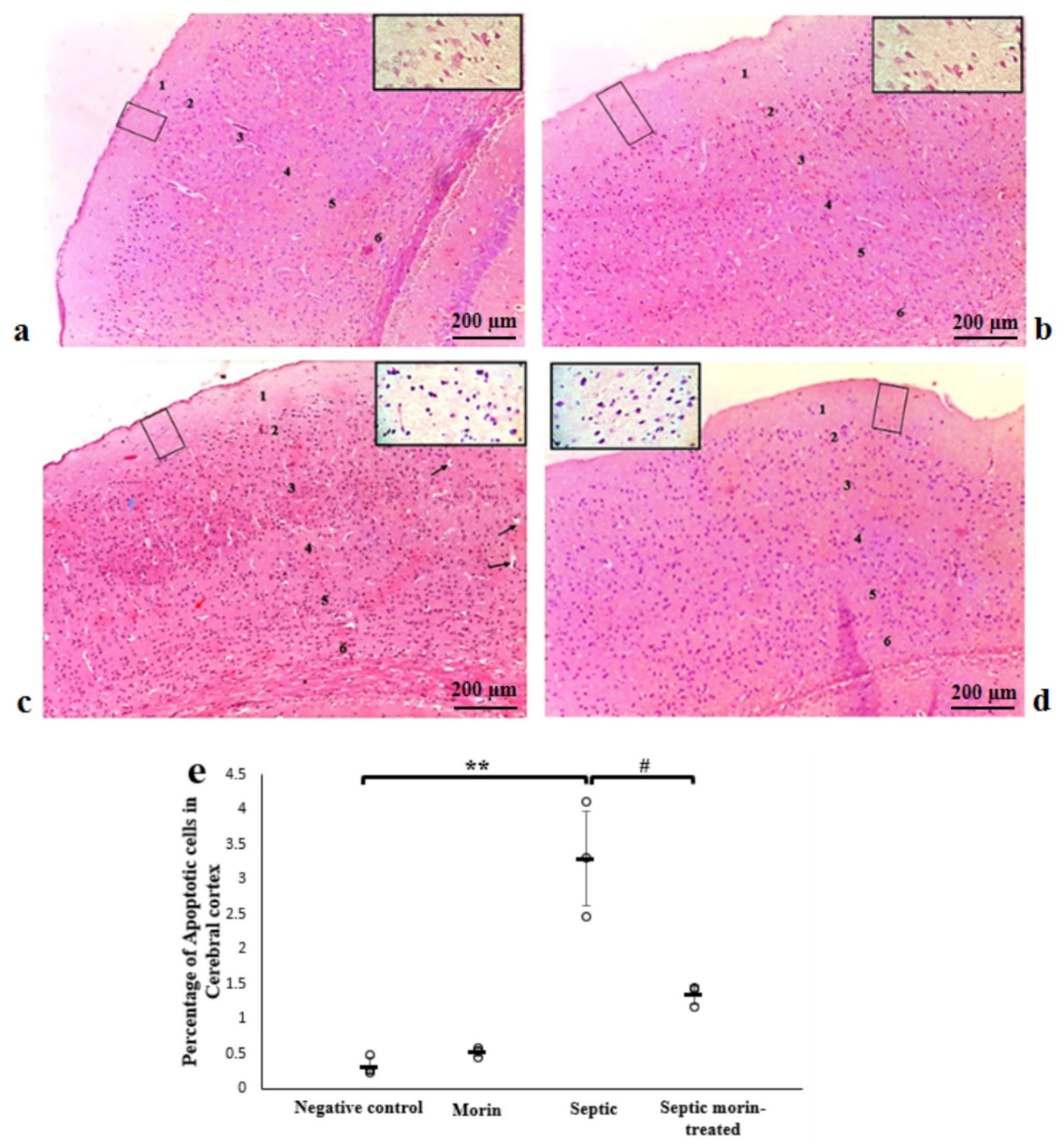
Photomicrographs of cerebral cortex showing; (**a**) Negative control group, showing the normal architecture of the cerebral cortex with its six layers (inset); (1) molecular layer, (2) outer granular layer, outer pyramidal layer (3), inner granular layer (4), inner pyramidal layer (5) and polymorphic layer (6). Note the presence of few numbers of neurons in the molecular layer (rectangle). (**b**) Morin group, showing the normal architecture of the cerebral cortex with its six layers (inset); (1) molecular layer, (2) outer granular layer, outer pyramidal layer (3), inner granular layer (4), inner pyramidal layer (5) and polymorphic layer (6). Note the presence of few neurons in the molecular layer (rectangle). (**c**) Septic group, showing multifocal histological changes in all layers of the cortex, including; multicellular outer granular (2) and multicellular outer pyramidal layers (3), while other layers (4, 5, and 6) are less crowded with cells. Note the presence of few numbers of neurons in the molecular layer (rectangle), shrunken nerve cells lost their processes, and had precellular halo (arrows), deeply stained neuron cell (blue arrow), faintly stained granular cell (red arrow) (inset). (**d**) Septic morin-treated group, showing improvement of the architecture of the six cortical layers (inset). Note the presence of few numbers of neurons in the molecular layer (rectangle). (Hx & E, X100) (**e**) Representative histological damage and quantitative analysis of apoptotic cells in sections. All data are expressed as mean ± SD. Circles represent raw data; horizontal lines represent mean for each group. (*) Symbol represents significance compared with normal group, where (*): *p* < 0.05 and (**): *p* < 0.001. (#) Symbol represents significance compared with septic group, where (#): *p* < 0.05 and (# #): *p* < 0.001

In the negative control group, as well as in morin-treated group (Fig. 8a and b), the histological features of the cerebral cortex layers revealed apparent intact well-organized neurons and glial cells in the different layers. These layers one: molecular layer, outer granular layer, outer pyramidal layer, inner granular layer, inner pyramidal layer and polymorphic layer. Cerebral cortex of septic group revealed multifocal histological changes in all layers of the cortex (Fig. 8c). Some areas became more cellular especially in the outer granular and outer pyramidal layer, while others were less crowded with cells. Most nerve cells were shrunken, lost their processes, and had pericellular halos. The pyramidal cells were more affected; they were deeply stained and became irregular in shape. Most of granule cells were affected in this layer and became faintly stained (Fig. 8d). 2-way ANOVA revealed a significant interaction between morin treatment and LPS on apoptosis in cerebral sections (*F*_1,8_ = 18.86, *p* < 0.05). Post-hoc pairwise comparisons, with Bonferroni correction, showed that there was a significant effect of LPS on apoptosis in cerebral sections compared with the negative control group (Bonferroni-adjusted *p* < 0.001). The cerebral cortex of septic morin-treated group showed improvement in nerve cells in many areas. Most of the pyramidal and granule cells were more or less normal in their architecture. Morin treatment demonstrated a significant effect compared with the LPS group (Bonferroni-adjusted *p* < 0.05) (Fig. 8e).

#### Hippocampus

**Fig. 9:**
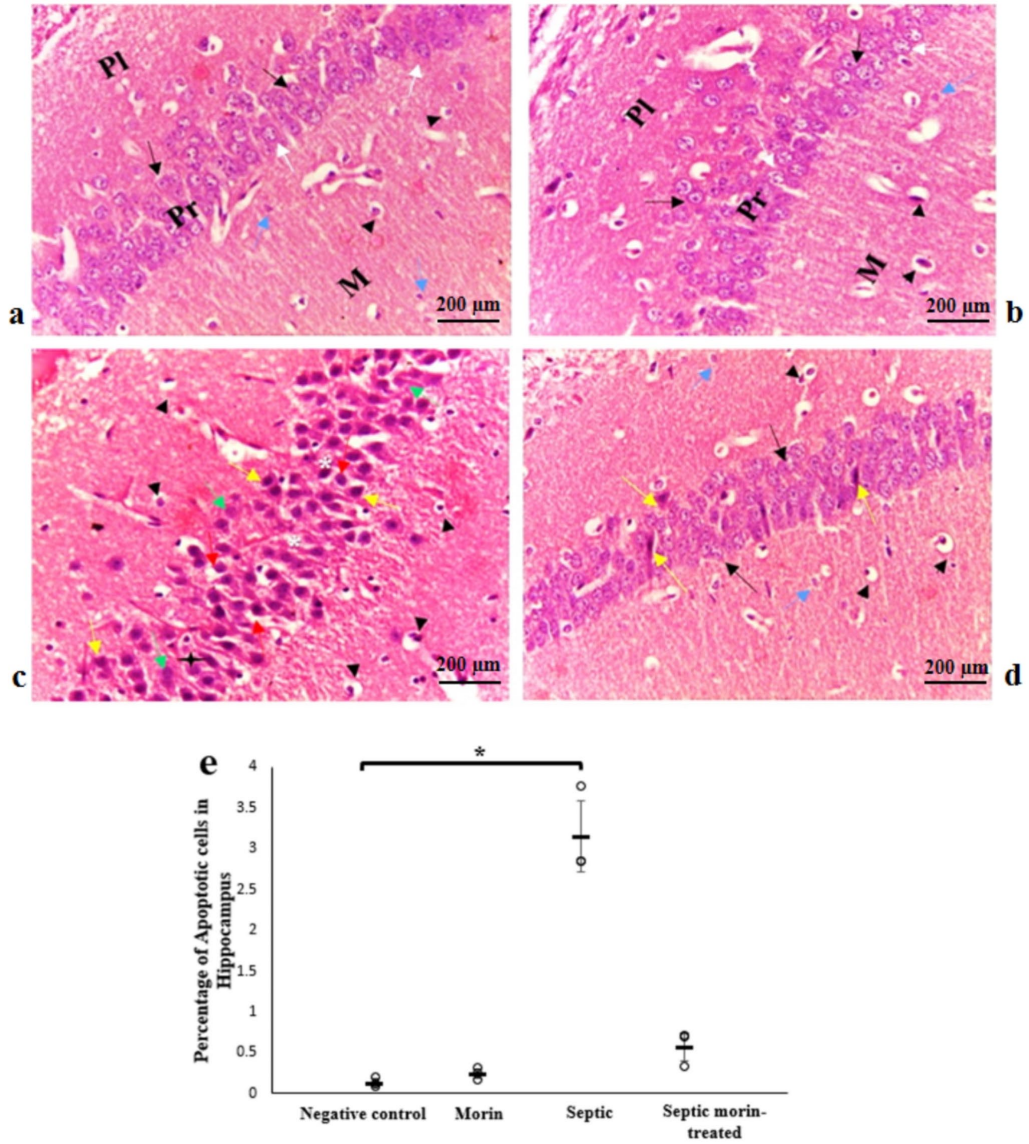
Photomicrographs of Amon’s horn area CA of hippocampus showing three layers; molecular (M), pyramidal (Pr), and polymorphic (Pl) layers. (**a**) Negative control group, (**b**) morin group, (**c**) Septic group, (**d**) Septic morin-treated group. Pyramidal cells with rounded vesicular nuclei (arrow), binucleated nuclei (white arrow), pyknotic nuclei (yellow arrow) and karyolitic nuclei (green arrow), glial cells (head arrow), interneurons (blue arrow), empty spaces (*), and wide perineuronal spaces (red arrow) are seen. (Hx & E, X400) (**e**) Representative histological damage and quantitative analysis of apoptotic cells in sections. All data are expressed as mean ± SD. Circles represent raw data; horizontal lines represent mean for each group. (*) Symbol represents significance compared with normal group, where (*): *p* < 0.05 and (**): *p* < 0.001. (#) Symbol represents significance compared with septic group, where (#): *p* < 0.05 and (# #): *p* < 0.001

In normal group, as well as in morin-treated group (Fig. 9a and b), the histological features of hippocampus demonstrated normal histological features of hippocampal regions with well-organized neurons in different layers with obvious large nuclei, and intact intercellular neuropil. In septic mice (Fig. 9c), signs of degeneration were evident, these changes comprised empty areas in the pyramidal layer, shrunken pyramidal cells with pyknotic nuclei, and wide perineuronal spaces. Molecular and polymorphic layers showed marked increase in the number of glial cells. In septic morin-treated group (Fig. 9d), cellular profile was nearly as in the negative control group, where few shrunken cells with pyknotic nuclei were rarely seen. Glial cells, interneurons and pyramidal cells appeared with vesicular nuclei. The Kruskal-Wallis test indicated significant differences between groups on apoptosis in hippocampus (*H*_3_ = 9.974, *p* < 0.05). After applying the Bonferroni correction, pairwise comparisons revealed that the LPS group had a significantly increased on apoptosis in hippocampus compared with the negative control group (adjusted *p* < 0.05). However, the septic morin-treated group did not show a significance compared with the LPS group (adjusted *p* > 0.05) (Fig. 9e).

### Immunohistochemistry

#### TNF-α

**Fig. 10:**
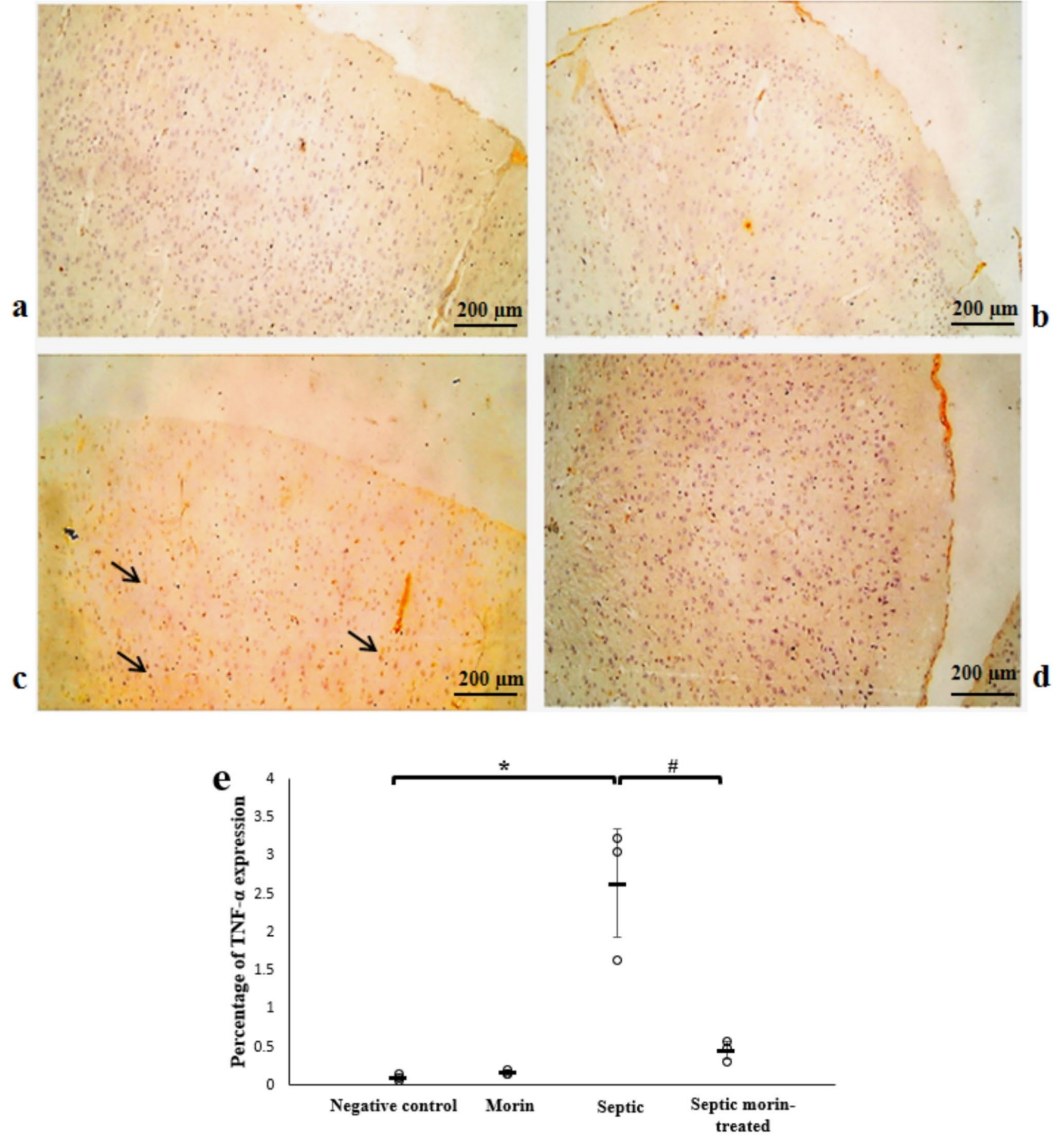
Photomicrograph showing immune expression of the TNF-α marker in the cerebral cortex of the different groups. (**a**) Normal control group, (**b**) Morin group, (**c**) Septic group, (**d**) Septic morin-treated group. Arrows point to positive cells with immune expression of the TNF-α marker. (100X) (**e**) Representative protein expression and quantitative analysis of TNF-α marker in the cerebral cortex of the different sections. All data are expressed as mean ± SD. Circles represent raw data; horizontal lines represent mean for each group. (*) Symbol represents significance compared with normal group, where (*): *p* < 0.05 and (**): *p* < 0.001. (#) Symbol represents significance compared with septic group, where (#): *p* < 0.05 and (# #): *p* < 0.001

The TNF-α immunohistochemical reaction showed almost no expression in the cerebral cortex of both the negative control and morin groups. In contrast, strong immune expression of TNF-α was observed in the layers of the cerebral cortex in the septic group compared with the negative control group. However, in the morin-treated group, the TNF-α immune reaction showed a negative expression compared with the septic group. 2-way ANOVA revealed a significant interaction between morin treatment and LPS on the expression of TNF-α (*F*_1,8_ = 19.41, *p* < 0.05). Post-hoc pairwise comparisons, with Bonferroni correction, showed that there was a significant effect of LPS on the expression of TNF-α compared with the negative control group (Bonferroni-adjusted *p* < 0.05). Additionally, morin treatment demonstrated a significant effect compared with the LPS group (Bonferroni-adjusted *p* < 0.05) (Fig. 10).

#### Cleaved Caspase 3

**Fig. 11:**
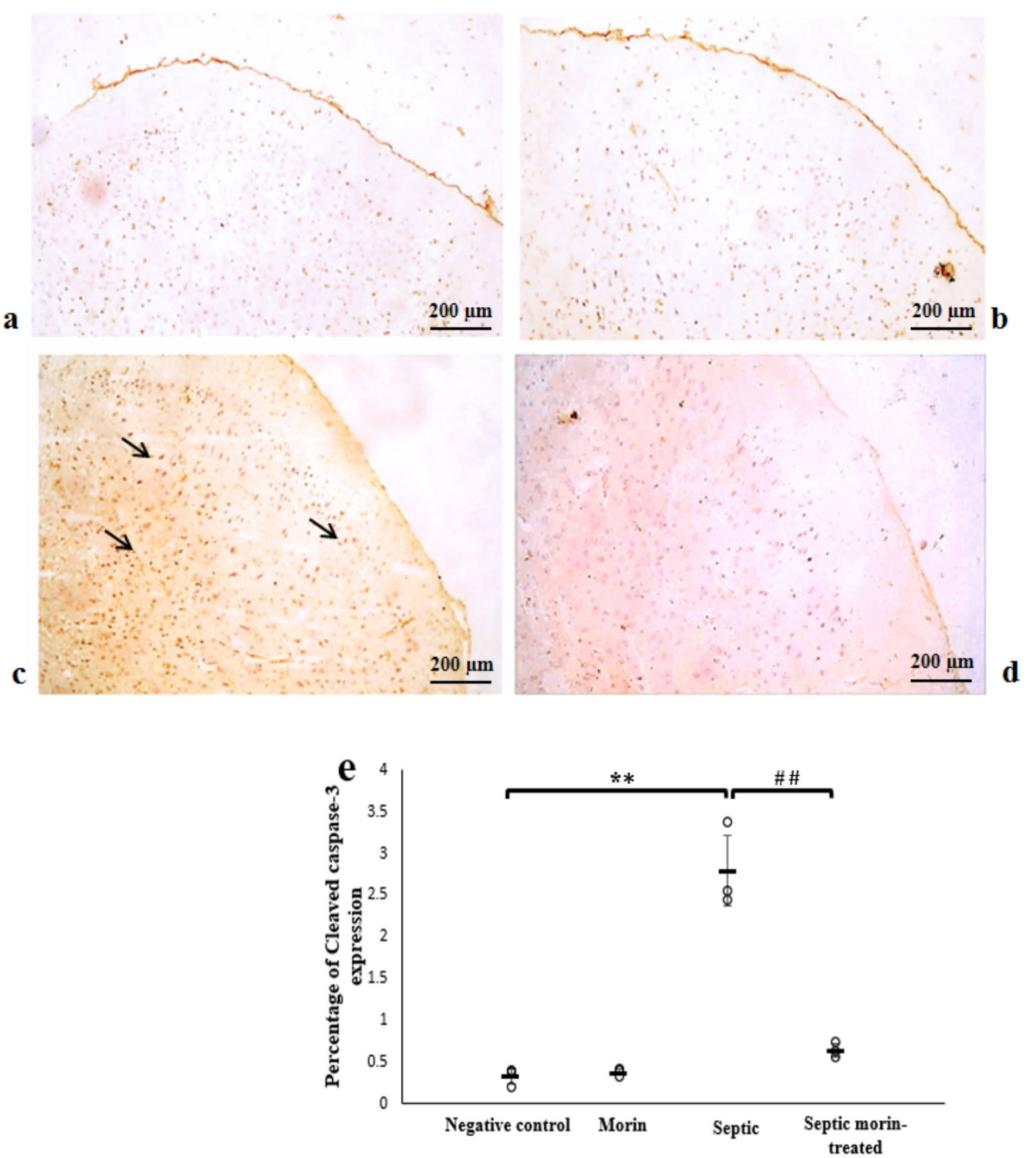
Photomicrograph showing immune expression of the cleaved caspase-3 marker in the cerebral cortex in the different groups. (**a**) Negative control group, (**b**) Morin group, (**c**) Septic group, (**d**) Septic morin-treated group. Arrows point to cleaved caspase-3-positive cells. (100X) (**e**) Representative protein expression and quantitative analysis of cleaved caspase-3 marker in the cerebral cortex of the different sections. All data are expressed as mean ± SD. Circles represent raw data; horizontal lines represent mean for each group. (*) Symbol represents significance compared with normal group, where (*): *p* < 0.05 and (**): *p* < 0.001. (#) Symbol represents significance compared with septic group, where (#): *p* < 0.05 and (# #): *p* < 0.001

No cleaved caspase-3 labeling was observed in the cerebral cortex area of both the negative control and morin sections. In contrast, marked immunoreactivity of cleaved caspase-3 was observed in some cells localized in the layers of the cerebral cortex in the septic group compared with the negative control group. However, in the septic morin-treated group, the cleaved caspase-3 immune reaction showed a negative compared with the septic group. 2-way ANOVA revealed a significant interaction between morin treatment and LPS on the expression of Cleaved Caspase-3 (*F*_1,8_ = 51.84, *p* < 0.001). Post-hoc pairwise comparisons, with Bonferroni correction, showed that there was a significant effect of LPS on the expression of Cleaved Caspase-3 compared with the negative control group (Bonferroni-adjusted *p* < 0.001). Additionally, morin treatment demonstrated a significant effect compared with the LPS group (Bonferroni-adjusted *p* < 0.001) (Fig. 11).

#### GFAP

**Fig. 12:**
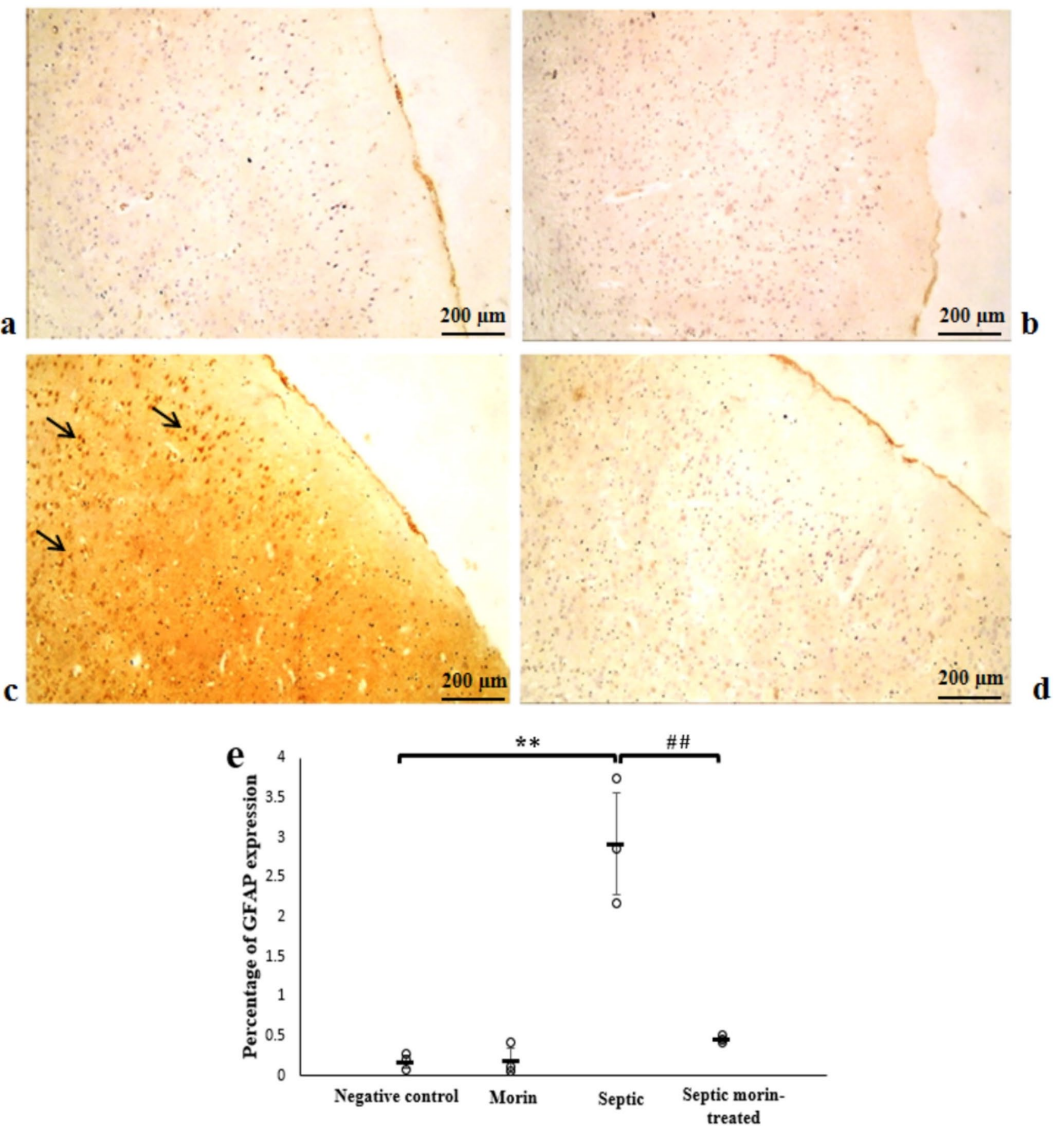
Photomicrograph showing immune expression of the GFAP marker in the cerebral cortex of the different groups. (**a**) Negative control group, (**b**) Morin group, (**c**) Septic group, (**d**) Septic morin-treated group. Arrows point to GFAP positive cells. (100X) (**e**) Representative protein expression and quantitative analysis of GFAP marker in the cerebral cortex of the different sections. All data are expressed as mean ± SD. Circles represent raw data; horizontal lines represent mean for each group. (*) Symbol represents significance compared with normal group, where (*): *p* < 0.05 and (**): *p* < 0.001. (#) Symbol represents significance compared with septic group, where (#): *p* < 0.05 and (# #): *p* < 0.001

The negative and morin groups showed no immune expression of GFAP in the cerebral cortex. In contrast, the septic group exhibited strong positive expression of GFAP immune reaction in the layers of the cerebral cortex compared with the negative control group. However, the sepsis morin-treated group showed negative reaction in the layers of the cerebral cortex compared with the septic group. 2-way ANOVA revealed a significant interaction between morin treatment and LPS on the expression of GFAP (*F*_1,8_ = 27.23, *p* = 0.001). Post-hoc pairwise comparisons, with Bonferroni correction, showed that there was a significant effect of LPS on the expression of GFAP compared with the negative control group (Bonferroni-adjusted *p* < 0.001). Additionally, morin treatment demonstrated a significant effect compared with the LPS group (Bonferroni-adjusted *p* < 0.001) (Fig. 12).

## Discussion

Sepsis is a severe systemic inflammatory response syndrome triggered by various infectious factors [25]. This condition leads to impaired blood brain barrier, which in turn causes brain damage, characterized by cognitive impairment and behavioral defects, referred to as sepsis associated encephalopathy (SAE) [26, 27].

In the current study, SAE was experimentally established by lipopolysaccharides injection. It was manifested by a significant decrease in the survival rate of septic mice, decrease in cognitive functions such as anxiety, a significant negative effect on spatial learning and memory, in addition to increases in depressive-like behavior in the surviving mice. Structurally, these symptoms were associated with histological alterations in the cerebral cortex, as well as in the hippocampus. Moreover, these manifestations were accompanied with a remarkable increase in the MDA content, and decrease in the total anti-oxidant capacity of the brain tissue. These pathophysiological alterations were confirmed immunohistochemically by increased brain tissue levels of GFAP, TNF-α, and cleaved caspase-3 following LPS injection.

The pathogenesis of SAE involves a multifactorial process characterized by an increase in oxidative stress, inflammatory response, and apoptosis [28, 29]. Oxidative stress, defined as an imbalance between the generation of reactive oxygen species and anti-oxidant defense capacity of the body, serves as a common final pathway related to inflammation, ischemia-reperfusion, multiple organ failure, and sepsis [30]. It also aggravates the expression of pro-inflammatory cytokines and apoptosis in the pathogenesis of SAE. In the present study, SAE mice exhibited a remarkable decrease in TAC and increase in the MDA content as an indicators of lipid oxidative damage. The observed alteration in sphingolipid metabolism is a potential mechanism of the LPS-induced BBB destruction [31, 32]. The current results also showed that SAE mice had decreased total anti-oxidant capacity. This is in agreement with the results of [33], which suggests that sepsis induces an imbalance between cellular anti-oxidant and oxidative states [34].

Oxidative stress is closely associated with sepsis and septic complications such as learning and memory deficits [30]. In the present work, septic mice suffered from decreased cognitive functions such as anxiety, which was evidenced by decreased locomotor activity in the open field and elevated plus maze tests. This is in agreement with the results of [35], who reported that infections and inflammatory processes may be causative factors in emotional disorders. Moreover, a significant negative effect of sepsis on spatial learning and memory was observed in the novel object recognition of the present study [36]. reported that; while an acute inflammation may prompt microglia activation that is beneficial in defending an individual against foreign invaders, chronic inflammation may have detrimental effects that result in neuronal losses and the onset of cognitive impair and physiological damage [37]. Furthermore, the current results showed decreased levels of exploration and lacking of recognition in the T-maze test [38, 39]. referred learning and memory dysfunction to brain tissue oxidative damage caused by deleterious effects of LPS. Increases in depressive-like behavior observed in the tail suspension test, is in link with the results of [40], who showed that some forms of depression can be viewed as a microglial disease. Indeed, microglial activation and the involvement of brain inflammatory cytokines are implicated in both depression and memory deficits [41, 42].

The current histopathological findings strongly supported the behavioral and biochemical findings. In the present work, histological sections of cerebral cortex and hippocampus demonstrated substantial differences among mice in negative control and septic groups. Sepsis induced histological changes in all layers of the cortex, as well as in the hippocampus, in addition to neuronal death, and loose of arrangement in the cells of hippocampus. These structural changes are presumably associated with reduced cognitive function, indicating a potential connection to the inflammatory response occurring both systemically and in the brain [43]. These findings were supported by the immunohistochemical results that showed increased expressions of cytokines, which may signify the disruption of the BBB integrity, leading to the infiltration of immune cells into the central nervous system. In fact, elevated levels of cytokines not only play a crucial role in inducing inflammatory reactions, but also serve as prognostic indicators [44]. In a recent study, increased cytokine levels including TNF-α was identified as an indicator of poor prognosis and adverse outcomes in sepsis-induced brain dysfunction [44, 45]. In the current work, numerous cleaved caspase-3-positive cells were observed. In addition, tissue GFAP level was significantly increased, which serves as a distinctive marker of reactive astrocytes, and this cytoskeletal protein contributes to the formation of a glial scar, which acts as a barrier facilitating the restriction of axonal extension [46, 47].

In the current study, surviving septic mice suffered from symptoms of brain disorder, such as hypo-activity, limp tail, hind leg paralysis, tetraparesis and moribund which are evidences of encephalomyelitis [43, 48]. Most of the mice suffered from closed eyes, and slightly ruffled fur, in addition to musty smell [49].

In the present study, the survival rate of septic group was significantly lower compared to other groups. Several studies revealed that the increase in mortality rate could be attributed to the profound hypotension and reduced cardiac output, which in turn lead to irreversible organ system failure and ultimately result in death [50, 51].

SAE is conventionally treated by antibiotics, anti-inflammatory drugs, sedatives, fluid therapy and vasoactive drug [52]. Despite the array of treatments available, there is currently no definitive therapy for SAE. Additionally, some of the existing medical interventions aimed at reducing mortality in sepsis and acute respiratory distress syndrome may be linked to neurological morbidity [53]. Consequently, there remains a pressing need for potential therapies that could effectively alleviate sepsis-associated encephalopathy.

Morin, a flavonol, has demonstrated a robust neuroprotective effect, attributed to its anti-inflammatory, antioxidant, and antimicrobial properties [54]. So, morin was chosen in this study to investigate its potential role to ameliorate sepsis-associated encephalopathy, and to assess the mechanism of its action. In the current study, morin ameliorated LPS-induced sepsis, and attenuated cerebral ischemia-induced oxidative damage in mice hippocampus and cerebral cortex. These positive effects were most likely through improving related biochemical markers, which were demonstrated by the values of MDA and TAC. Therefore, morin might had downregulated the expression of pro-inflammatory cytokines, and prevented the effects of sepsis-induced cognitive and behavior impairment and improved the spatial learning and memory capacity of septic mice. A better survival rate was also observed. These results were confirmed by the attenuation of neuronal apoptosis and microglial activation, and improvement of cognitive and behavior after morin treatment.

SAE mainly depends on the occurrence of oxidative stress and the consequent excessive inflammatory response [2]. Morin, known for its anti-oxidant properties, is affirmed herein by its capacity to counteract SAE-induced raise in the MDA and the decline in the TAC [55, 56]. Moreover, morin intervention effectively downregulated the expression of pro-inflammatory cytokines mediated through its anti-inflammatory activity [57, 58]. Additionally, morin showed anti-apoptotic effect through decreasing expression of cleaved caspase-3, and exerted neuro-protective action through decreasing lipid peroxidation. These observations may explain the improvement in both the histological and immunohistochemical results.

## Conclusion

In conclusion, the administration of morin demonstrated a beneficial impact on LPS- induced sepsis associated with encephalopathy in an experimental model of adult male albino mice. So, it is recommended that morin could serve as a promising antioxidant supplement, particularly in areas prone to LPS contamination, offering a significant protective medicinal option against neuro-toxicity. However, due to the mild degenerative changes still observed in some cells in this study, further intensive experimental and clinical investigations may be necessary to refine the dosage and duration of morin treatment.

## Data Availability

Data is provided within the manuscript.
